# Toward autonomous robotic prostate biopsy: a pilot study

**DOI:** 10.1007/s11548-021-02437-7

**Published:** 2021-07-05

**Authors:** Bogdan Maris, Chiara Tenga, Rudy Vicario, Luigi Palladino, Noe Murr, Michela De Piccoli, Andrea Calanca, Stefano Puliatti, Salvatore Micali, Alessandro Tafuri, Paolo Fiorini

**Affiliations:** 1 Department of Computer Science, University of Verona, Verona, Italy; 2 Department of Urology, University of Modena and Reggio Emilia, Modena, Italy; 3 Department of Urology, University of Verona, Verona, Italy

**Keywords:** Medical robotics, Prostate biopsy, Automatic segmentation, Image fusion, Robot-assisted biopsy

## Abstract

****Purpose**:**

We present the validation of PROST, a robotic device for prostate biopsy. PROST is designed to minimize human error by introducing some autonomy in the execution of the key steps of the procedure, i.e., target selection, image fusion and needle positioning. The robot allows executing a targeted biopsy through ultrasound (US) guidance and fusion with magnetic resonance (MR) images, where the target was defined.

****Methods**:**

PROST is a parallel robot with 4 degrees of freedom (DOF) to orient the needle and 1 DOF to rotate the US probe. We reached a calibration error of less than 2 mm, computed as the difference between the needle positioning in robot coordinates and in the US image. The autonomy of the robot is given by the image analysis software, which employs deep learning techniques, the integrated image fusion algorithms and automatic computation of the needle trajectory. For safety reasons, the insertion of the needle is assigned to the doctor.

****Results**:**

System performance was evaluated in terms of positioning accuracy. Tests were performed on a 3D printed object with nine 2-mm spherical targets and on an anatomical commercial phantom that simulates human prostate with three lesions and the surrounding structures. The average accuracy reached in the laboratory experiments was $1.30 \pm 0.44\, \text {mm}$ in the first test and $1.54 \pm 0.34\, \text {mm}$ in the second test.

****Conclusions**:**

We introduced a first prototype of a prostate biopsy robot that has the potential to increase the detection of clinically significant prostate cancer and, by including some level of autonomy, to simplify the procedure, to reduce human errors and shorten training time. The use of a robot for the biopsy of the prostate will create the possibility to include also a treatment, such as focal ablation, to be delivered through the same system.

## Introduction

Prostate cancer (PCa) is the second most common cancer, after breast cancer, and is one of the leading causes of death in men [11]. Early and reliable detection of PCa has a huge impact on the successful treatment of high-risk patients and on avoiding overtreatment in low-risk patients. The most reliable technique to detect PCa and to estimate its aggressiveness is needle biopsy [14]. Biopsies are carried out most often under US guidance. Traditional transrectal biopsy is replaced by the safer transperineal biopsy, which ensures lower infection risks, but may require sedation and must be performed in the operating room. A way to simplify the transperineal procedure and to reduce patient trauma is to use a small number of entry points, from which to reach all the areas of interest; this procedural improvement, however, is highly dependent on the experience of the doctor.

The PROST robotic system aims at reducing human error in biopsy planning and execution. PROST will add a measure of autonomy to the procedure by using Machine Learning (ML) algorithms during the planning phase (i.e., target selection in the pre-operative MR, fusion of MR with real-time US, entry point planning) and in the execution phase by autonomously positioning a needle guide while tracking the position of the US transrectal probe. Nevertheless, the approval of the target position is done by the user and the insertion of the needle is manual so that the doctor has the complete control of the surgical task.

According to the classification proposed in [15], there are six levels of autonomy for medical robots:

Level 0: No autonomy
Level 1: Robotic assistance
Level 2: Task autonomy
Level 3: Conditional autonomy
Level 4: Supervised plan/execution of a full task
Level 5: Full autonomy

The PROST prototype we describe here is positioned at autonomy level 1. The device and the human share decisions and actions; the device autonomously provides both cognitive and manual assistance to the human operator. PROST offers cognitive support during target acquisition and entry point planning, whereas manual assistance consists in facilitating the accurate positioning of the biopsy needle. These functions will allow increasing the procedure’s accuracy independently of the expertise level of the operating physician.

Prostate biopsy is commonly performed by urologists as a freehand procedure. The most advanced techniques use a pointing device to orient the needle and correlate its position to the US image; a US transducer is mounted on a stepper that allows for translation and rotation. We review below the main commercial products and some state-of-the-art prototypes related to prostate biopsy:

- *Uronav* (Philips Medical Systems, Netherlands) is an MR/US-guided fusion biopsy system that fuses pre-biopsy MR images of the prostate with real-time US images.
- *Koelis Trinity* (Koelis, Meylan, France) is a freehand automated fusion biopsy system for 3D trans-perineal approach. It uses a dedicated 3D SideFire US probe, patented methods for image fusion [7] and a range of smart guides.
- *Real-Time Virtual Sonography (RVS)* (Hitachi, Tokyo, Japan) and *Virtual Navigator* (Esaote, Genoa, Italy) are commercial fusion systems that can be integrated in an outpatient room.
- *BK Fusion* (Analogic, Peabody, MA, USA) is a fusion system that extends BK US machines with fusion for MR/US registration.
- *Artemis* (Eigen, Grass Valley, CA, USA) is a semi-robotic arm with encoders for tracking the US probe used to navigate the prostate in real-time and to precisely position the biopsy needle.
- *iSR’obot MonaLisa* (Biobot Surgical LTD, Singapore) combines a robotic arm with image fusion technology.
- Recently, the prototype of a robotic system for transrectal prostate biopsy was presented in [8]. The system implements the procedure under US guidance and has already been tested on humans.

A further study on systematic robot-assisted biopsy was presented by Han et al. in [2]. The authors employed a robot for transrectal US biopsy previously described in [12].

All the systems briefly described above have some automatic functions, whose operation must be integrated with the human operator’s actions. Biopsy planning is done statically (e.g., in Artemis, MonaLisa) through fusion and target selection; the systems that update the prostate’s position dynamically (e.g., Koelis) do not include dedicated hardware to update the planning, relying only on the experience of the user. By integrating ML techniques for real-time prostate segmentation with robotic hardware, the solution we are presenting here will advance the autonomy of the preceding solutions by offering task replanning in response to perceived changes and so improving the accuracy in target selection.

**Fig. 1:**
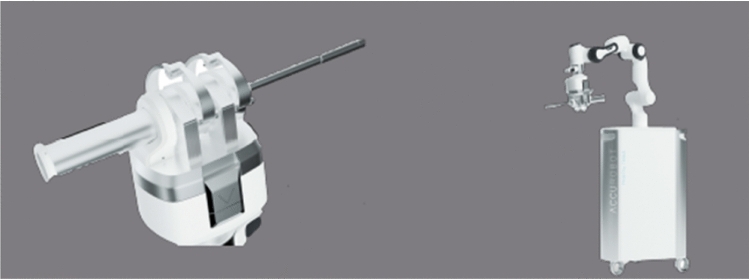
Artist rendering of the PROST system. Left: the robotic head, used for biopsy procedures. Right: the head as a component of a larger robotic system, used for more advanced procedures. In this paper, we describe the robotic head, which can be used independently of the base

**Fig. 2:**
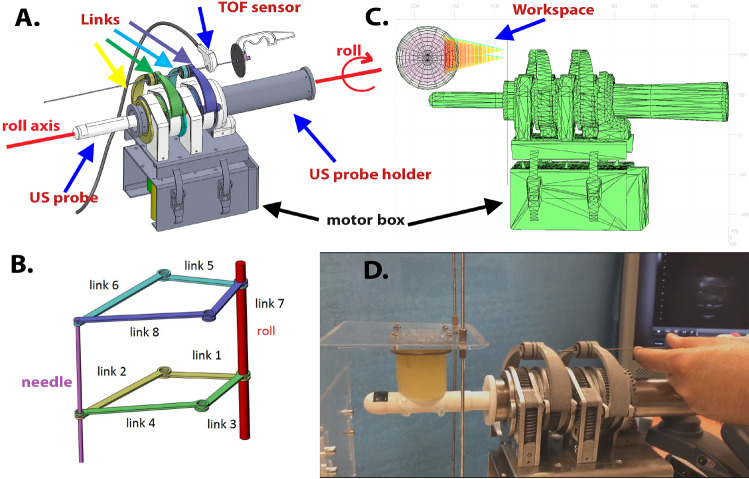
**a**: PROST CAD shown with the US probe. The probe is actuated to rotate on the sagittal plane (roll). A TOF sensor measures the depth of the needle insertion. **b**: Schematic view of the SCARA structure. Each pair of same-color links is actuated by one of the motors. **c**: PROST’s workspace is a cone with apex at the insertion point. **d**: PROST prototype with the synthetic phantom during a needle insertion test

Our system is designed for precise targeting of the prostate under ultrasound (US) guidance (Fig. 1) in transperineal biopsy procedures. PROST integrates target selection, image fusion and biological motion compensation. We show in synthetic tests that PROST allows physicians with little experience with needle biopsy to reach the same level of accuracy as expert urologists. PROST allows reaching the entire prostate gland through just two punctures. These act as pivot points, resulting in a conical configuration of core positions (Fig. 2). Accuracy in orientation is even more important when the number of insertion points is low; instead of requiring excessive skill of the human, we leverage the robot’s potential for mechanical accuracy. The use of a lower number of insertion points can reduce trauma to the patient without sacrificing the accuracy of a template biopsy; it also leads to a quicker execution. We aim to increase the accessibility of high-quality prostate biopsy thorough compatibility with outpatient settings, quick set-up and fast intervention time independently of the expertise level of the operating physician. The use in outpatients’ clinics will potentially allow time and cost reduction in the procedure in comparison with template biopsy, which is currently carried out in the operating room.

In the next sections, we describe the hardware structure of the robot (“PROST robot” section), then the experimental setup (“Tests on synthetic phantom and Tests on anatomical phantom” sections). “Level of Autonomy and Automatic segmentation of the prostate” sections provide an overview of key elements of the PROST software, whose details are outside the scope of this paper, since these parts are subject to continuous updates. “Results” section presents the results of the tests, while “Conclusions and future work” section summarizes the paper and describes our plans for future developments of the system.

## Material and methods

### PROST robot

The robotic system is composed of two-joint arms that move along parallel planes (Fig. 2). They are coupled by an axis passing through the center of the two joints. This axis determines the needle orientation. Two small spheres with a hole, installed on each of the two joints, allow the needle to pass through the center of the joints. Each arm is composed of four links: the first one rotates around a common axis (roll axis in Fig. 2a and b). The dimensions of the links are as follows: links 1 and 3 are 40-mm long, links 2 and 4 are 61.5 mm long, links 5 and 7 are 42 mm long, and links 6 and 8 are 66 mm long. The transmission between the motors to the first joint is realized with an endless screw. The force and torque of the mechanism are influenced by the presence of this screw; however, the effect is not relevant because the movement of the links is unconstrained and there is no payload. Once the robot reaches the final position, nonreversible gears ensure that the desired orientation is maintained. The US probe axis coincides with the roll axis. With this configuration, the robot has two degrees of freedom (DOFs) for reaching the entry position, two DOFs for needle orientation and one DOF for the US probe. Altogether, there are 5 DOFs, corresponding to 5 motors.

The robot positions a cannula oriented toward the target; a biopsy needle then slides through the cannula at the entry point, directed toward the target. The needle insertion is done manually. The entry point is calculated by the PROST software as a fixed point that allows reaching all the selected targets. For each target point, the orientation is given as the line through the spheres in the top joints that connects with the entry point. A time of flight sensor (TOF) is attached to the cannula to measure the insertion depth of the biopsy needle. The calibration of the TOF with the robot ensures an overall error—evaluated at the needle tip—of less than one millimeter.

**Fig. 3:**
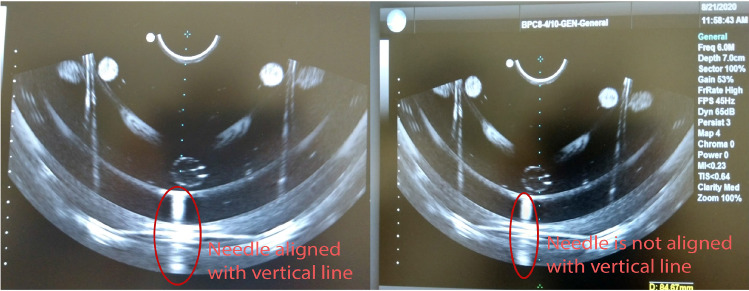
Left: The needle (the hypo-echogenic segment encircled in the figure) is aligned with the vertical plane, represented as a dotted line in the middle of the image. Right: The needle before alignment with the vertical plane

**Fig. 4:**
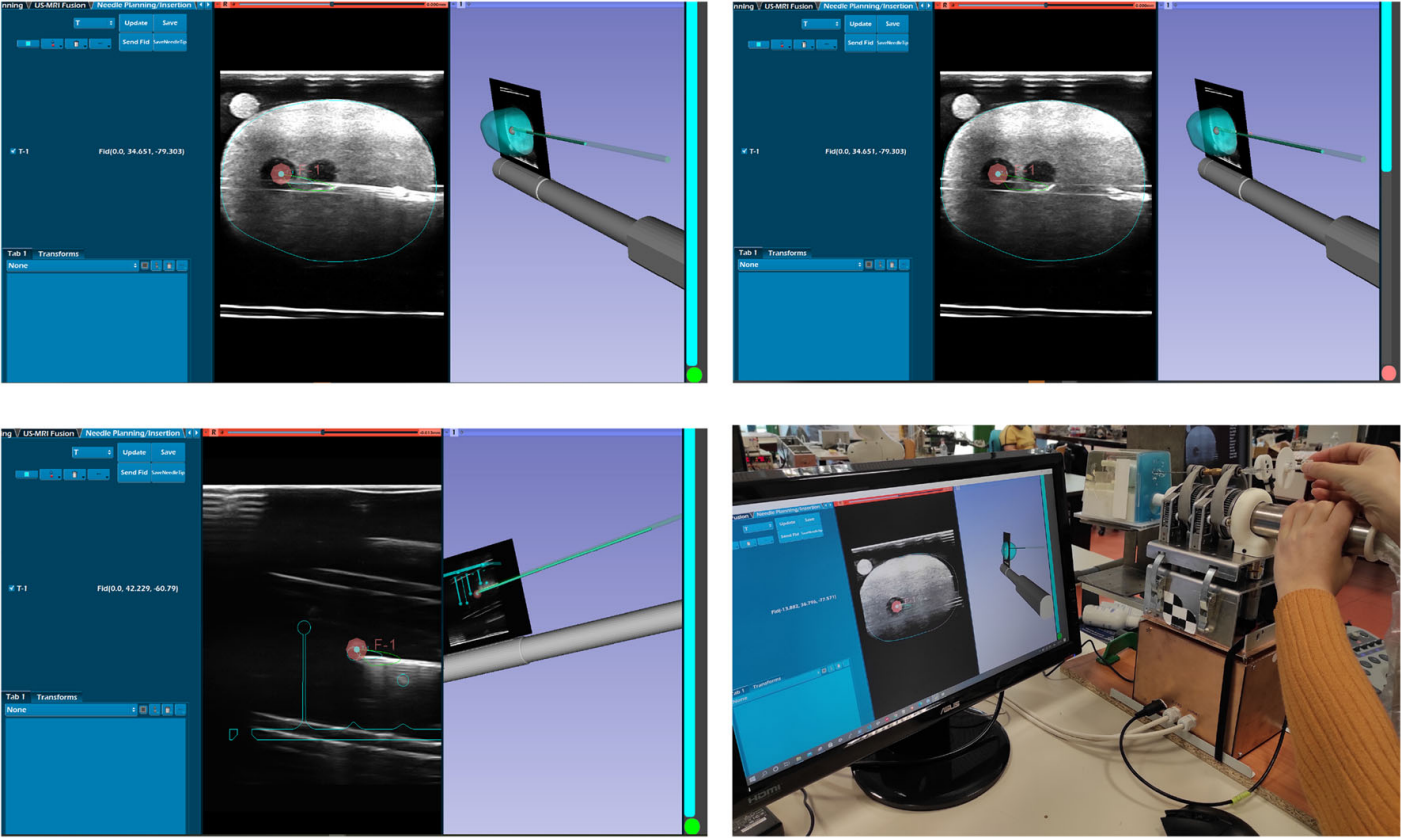
Graphical user interface and the biopsy procedure. Top left: US image of the anatomical phantom registered with MR contours during a needle insertion; the US image contains 2 targets, one of which is selected (in pink); the needle reached the target and can be seen in the US image as a hyper-echoic line; the whole procedure can be seen in 3D; on the right, a progress bar shows the distance to the target, represented by a green circle. Top right: before reaching the target, a green path shows the projected trajectory both in 2D and 3D. Bottom left: targeting the synthetic phantom. Bottom right: overall view of the system during the insertion in the anatomical phantom

The PROST prototype integrates the Ultrasonix US system with a compatible bi-planar US probe (BK Medical, Peabody, Massachusetts, USA); the system will be compatible with probes by different manufacturers. An initial calibration of the US image with the robot coordinate system is performed using the Plus toolkit [6]. Plus allows also for temporal calibration between US images and the robot tracking, so that 3D US image reconstruction is possible. Fine calibration is performed through a gel phantom by inserting the needle in the (vertical) plane at 0 degrees, as given by the robot kinematics. Since the probe is bi-planar, we first identify the needle in the transverse image and then rotate the probe until the sagittal plane aligns with the needle in the image (Fig. 3). We calibrate the position of the US image along the direction of insertion (defined as the *z*-axis in the robot’s coordinate frame) by computing the *z* coordinates of a needle when it touches the most anterior part of the US image. These coordinates, together with the dimensions of the pixel in the US image (as given by the US machine manufacturer), are transferred to the Plus software. Real-time communication with the image stream and the robot control employs the open-source software OpenIGTLink [13]. A dedicated user interface (Fig. 4) and image processing software, based on 3D Slicer and developed in Python, was developed in collaboration with our clinical partners.

### Level of autonomy

PROST is capable of autonomy “Level 1” because it provides assistance to the operator in the phases of target selection, planning, and biopsy execution. The cognitive assistance is particularly important in the selection of small targets that require a great expertise to be detected and eyes not affected by the fatigue of a day’s work. From the point of view of the procedure’s accuracy, the biopsy’s execution is of similar importance. Here, PROST provides assistance to the physician by accurately positioning the needle at the appropriate entry point and with the correct orientation. When PROST reaches the desired orientation, non-reversible gears fix it in place; all the physician has to do is slide the needle in the direction provided by the robot. The TOF sensor (see Fig. 2) allows the robot to detect when the right depth has been reached and triggers the display of a green circle.

### Tests on synthetic phantom

**Fig. 5:**
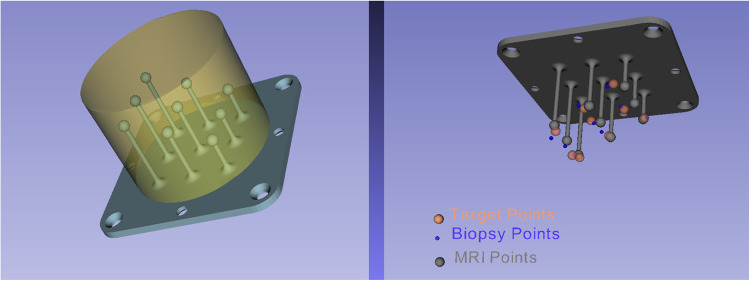
Left: 3D-printed phantom with 9 targets. The phantom is immersed in a silicon shell (yellow) transparent to US. Right: the ‘Target points’ are chosen by the user in the US images, CAD (CT or MR) points come from the 3D model, and the ‘Biopsy points’ are those reached by the tip of the needle during a robot-assisted puncture. The error is computed as the difference between ‘Target points’ and ‘Biopsy points’

We designed a synthetic phantom that includes 9 small spheres as targets, each with a diameter of 2 mm (Fig. 5). The distribution of the targets covers a volume similar to the volume of the prostate, and their small size allows for a fine measure of the accuracy of the PROST system . The geometric structure is 3D printed at high resolution and immersed in a silicon shell transparent to ultrasound waves.

The phantom was also acquired in CT (computed tomography) so that a 3D volumetric medical image can be available for image fusion. Alternatively, one can employ the 3D CAD file of the phantom’s surface (Fig. 5) previously used to print the structure, to provide an accurate map of the targets through image registration. The registration of the robot coordinate frame with the CT/CAD coordinate system is done by manually identifying the center of the targets in the US image and then using a point-to-point registration algorithm [9].

**Table 1:** Results of the tests on the synthetic and anatomical phantoms

	Synthetic phantom				Anatomical phantom			
Error(mm)	Robot		US		Robot		US	
	Mean	Std. Dev.	Mean	Std. Dev.	Mean	Std. Dev.	Mean	Std. Dev.
Expert	0.47	0.51	1.10	0.29	1.12	0.51	1.73	0.42
Users	0.83	0.65	1.57	0.79	0.80	0.64	1.43	0.49
	0.61	0.47	1.37	0.39	0.31	0.22	0.69	0.08
	0.99	0.75	1.52	0.50	1.24	0.62	1.81	0.49
	0.93	0.81	1.44	0.51	1.51	0.30	1.31	0.21
Non-	1.11	0.76	1.17	0.33	1.55	0.99	1.74	0.39
expert	0.94	0.79	1.37	0.66	1.59	0.07	1.61	0.54
Users	0.96	0.68	1.05	0.30	1.52	0.18	1.54	0.28
	1.20	0.85	1.21	0.29	1.97	0.79	1.79	0.22
	1.05	0.95	1.26	0.35	0.93	0.44	1.80	0.30
Average	0.91	0.72	1.30	0.44	1.25	0.48	1.54	0.34

The user interface allows the physician to choose targets in the US image (the Target Points in Fig. 5) and then send their coordinates to the robot, which then reaches the entry point and orients the needle in the desired configuration.

For each insertion, the error is measured as the distance between the needle tip (a Biopsy Point in Fig. 5) and the corresponding target. This distance measurement is done in two ways: one is in the US image and the other is in the reference frame of the robot. In the first case, when the needle tip and the target are in the same US plane, we compute the error independently of the robot kinematics, whereas when they are not in the same plane, we rotate the US probe from the target to the needle tip to compute the distance (Fig. 5).

The accuracy of the measurement in the first scenario (US error in Table 1) is influenced by the accuracy of the calibration between the US image and the robot, by the accuracy of the points’ identification in the image and by the mechanical accuracy of the robot. This measurement is closer to reality, since the resolution of the US image is high (between 0.2 and 0.5 mm), and the needle tip and the target are, most of the time, in the same planar image.

In the second scenario (Robot error in Table 1), the accuracy of the measurement is influenced by the accuracy of the calibration between the US image and the robot and the calibration between the needle tip and the robot through the TOF sensor. As shown in Table 1, this error is lower when compared to US error. The reason is that the error does not take into account the small deformations caused by the physical interaction of the needle with the phantom.

**Fig. 6:**
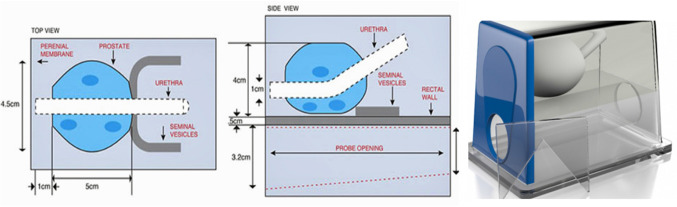
CIRS 053L commercial phantom. It includes: perineal membrane, prostate, urethra, seminal vesicles, lesions, and rectal wall

### Tests on anatomical phantom

We used the CIRS 053L Ultrasound Prostate Training Phantom (CIRS, Inc., Norfolk, USA), a multi-modality disposable phantom developed for practicing procedures which involve scanning the prostate with a rectal probe. The CIRS 053L phantom includes rectal wall, seminal vesicles, perineal membrane, urethra and 3 hypoechoic lesions of diameter approximately 10 mm (Fig. 6). The structures are visible under CT, MR, US and elastography.

For these tests, we segmented the lesions and the prostate from MR images, whereas the US images were segmented in real-time (see “Automatic segmentation of the prostate” section). The prostate segmentation allows for automatic registration that can also be manually adjusted. Since the phantom does not deform significantly, we employed the ICP registration algorithm [1]. The fusion of the two images allows mapping the lesions segmented in MR onto the robotic reference frame. The user then marks the center of the lesion in the MR image and PROST will automatically orient the needle. Insertion is then performed manually by the urologist.

### Automatic segmentation of the prostate

We implemented in PROST’s software [10] a proof-of-concept module for semantic segmentation of US scans of the anatomical CIRS phantom.

**Fig. 7:**
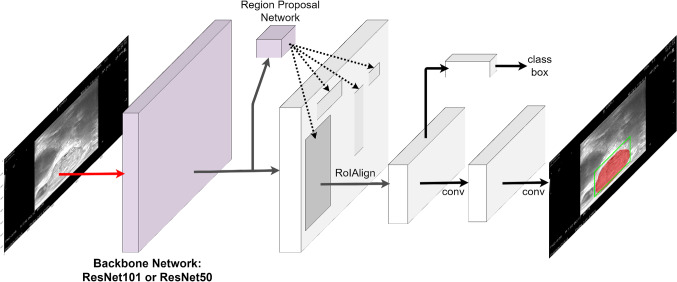
Architectural diagram for segmentation with detail on ResNet Backbone and Region Proposal Network for ROI selection. The last layers perform prostate segmentation

Based on this proof of concept method, we have tested a more complex neural network—this time on real human data. The network was inspired by the work presented in [3] and was adapted to our case. The architecture of the network extends the ResNet architecture [4] with layers that compute a region of interest (ROI) enclosing the prostate in a bounding-box of minimal area and with layers that perform the computation of the prostate mask (Fig. 7).

**Fig. 8:**
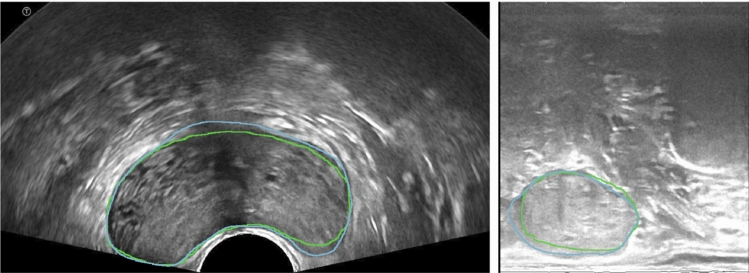
Segmentation of the prostate in US images, in axial (left) and sagittal (right) view: the blue line shows the ground truth, whereas the green line shows the result of automatic segmentation

This network pushed the accuracy (computed as Dice score) to 89% on phantom images and allowed segmenting the prostate regardless of the scanning plane (axial or sagittal) and regardless of sensor arrangement (linear or convex). In the case of real patient data, the segmentation accuracy was around 85% (Fig. 8).

## Results

**Fig. 9:**
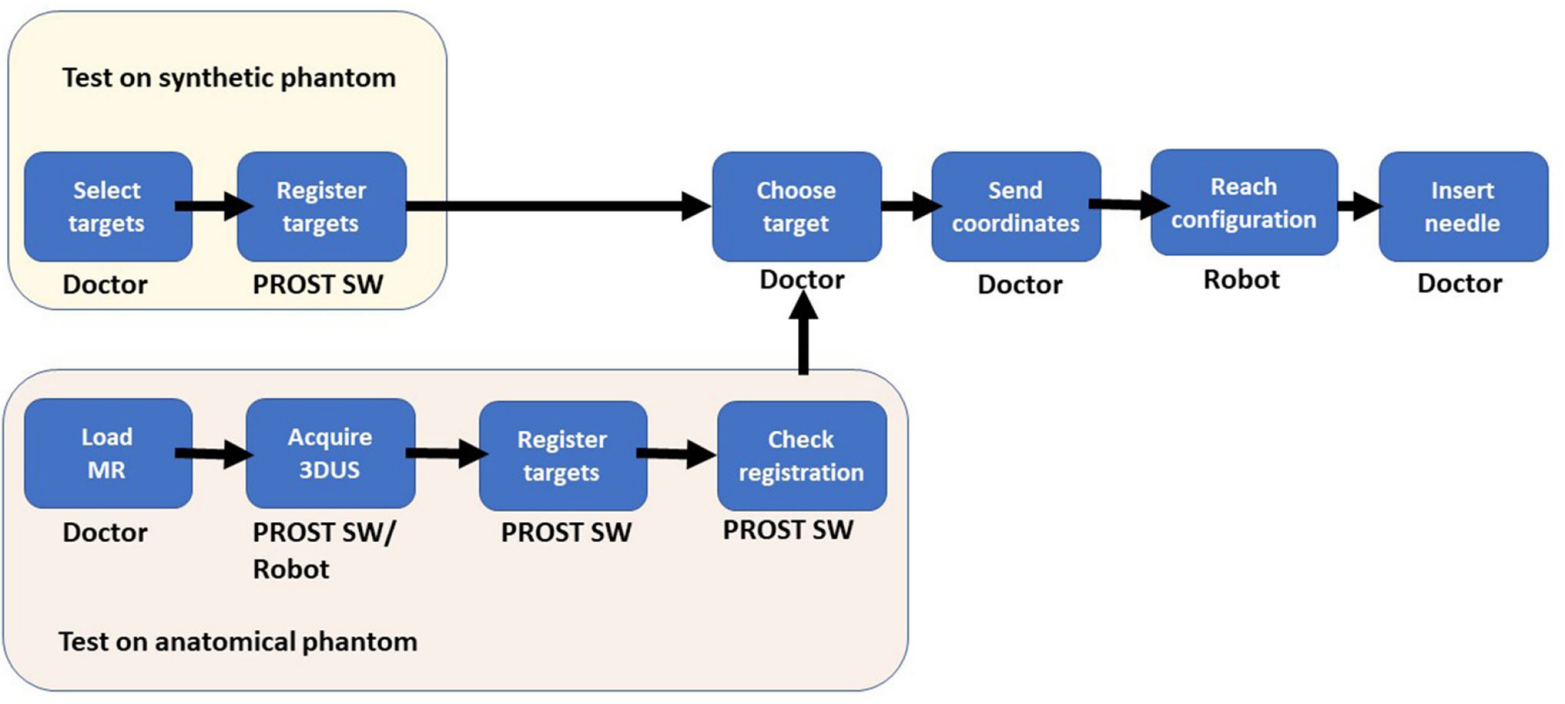
Workflow for phantom experiments: synthetic (top row); anatomical (bottom row)

A cohort of 10 urologists was involved in the study. Five were experts, with more than 100 prostate biopsies performed, and 5 inexperienced, with only 10-50 biopsies of experience. The time required for each insertion was less than 1 minute. The first test of each urologist took around 10 minutes, whereas the second test took less than 5 minutes, including the time required for the urologist to get used to the robot interface (Fig. 4). Figure 9 shows the workflow of the test on the synthetic phantom: the user manually selects the 9 targets by moving the US probe and the software registers them with the targets extracted from the CAD model. This registration serves only as reference to visually guide the targeting procedure (Fig. 4 bottom row left), in consideration of the symmetry of the phantom.

The workflow of the anatomical phantom test (Fig. 9) highlights how the robotic prostate biopsy works: the user loads the pre-operative MR data, the robotized US probe acquires the 3D US, targets from MR are registered in the robotic reference system, the doctor checks the registration result and chooses the target points. The robot moves accordingly (Fig. 4).

Table 1 reports the test results. In the synthetic phantom test, the points in the US image are different than the points on the registered structure, due to the registration error between the 3D CAD model and the US image. Nevertheless, in this experiment we chose the points from the US image as targets for needle insertion. The reason is that the synthetic phantom tests serve only to measure the accuracy of the robot mechanism and the calibration of the system. When the needle trajectory is distant from the axis of the conic workflow, the targeting error is larger. The average error we obtained for the synthetic phantom, considering all the trials and all the participants, was $0.91 \pm 0.72 mm$ in the reference frame of the robot and $1.30 \pm 0.44 mm$ in the US image. An obvious fact we noticed was that the error does not depend on the experience of the user, but on the precise preparation of the setup (calibration and identification of the target points).

The results on the anatomical phantom (Table 1) show little difference with the synthetic phantom tests. The main difference in the procedure is that here we chose the targets in the MR image. Even if the targets are visible in the US images, we wanted to replicate the setup of a real targeted fusion biopsy. The MR image is registered with the robot by using the segmentation of the whole prostate gland (“Tests on anatomical phantom” section) and the rigid registration algorithm. In this case, the registration error also accumulates with the previous sources of error, especially with the image calibration error. The average error, computed over all the participants and all trials, was $1.26 \pm 0.48 mm$ in the robot reference frame and $1.55 \pm 0.34 mm$ in the US image. By using the US image only we have an estimation of the error independent of the robotic system. The higher accuracy of the measurement in the reference frame of the robot is due to the rigid assumption, when if fact the needle may curve due to the bevel effect [5]. Again, we did not notice any significant differences between experienced users and non-experienced users. We also noticed a better accuracy and precision in the synthetic test as compared with the anatomical test (Table 1, last row). This is due to the fact that the in the synthetic phantom the structure was rigid (3D printed) and the targets were smaller. In this case, the needle could stop at the target, while in the anatomical phantom the target structures were soft and the needle could overshoot.

Our results prove the usability of PROST, regardless of user experience and with an accuracy of around 1 mm. Considering that clinically significant lesions tend to exceed 5 mm of diameter, the system allows collecting targeted biopsies for every lesion of the prostate.

## Conclusions and future work

In this paper, we presented the first prototype of PROST, a prostate biopsy robot that includes a level of autonomy in target identification, image fusion and needle guidance. PROST can guarantee that high-accuracy prostate biopsies are accessible, thanks to a low-cost solution that works independently of the clinician’s experience.

The PROST robotic system has several potential clinical advantages with respect to current products and laboratory prototypes: accurate targeting—comparable with MR guided biopsy but with the advantage of real-time US guidance and 3D visualization—repeatability of the biopsy for active surveillance by mapping all the locations of biopsy cores back into the MR image on file through robotic registration, standardization of the biopsy procedure regardless of the user’s experience level, less trauma for the patient, and the possibility of combining the robot with other diagnostic and therapeutic devices. The next step will be testing on cadavers, to verify the portability of this system to a clinical setting.

The design of a second prototype will take into account both objective parameters (robot workspace, movements of the mechanism, compatibility with clinical standards, software and hardware requirements) and subjective evaluations (e.g., ergonomics, usability, improved user interface) to reach the goal of a safer and more accurate prostate biopsy.

Particular attention will be paid to the sterilization constraints required of a surgical device. Small parts that are in contact with the needle, such as the robotic arms, will be removable and disposable, while the mechanical part will be detachable from the electronic part for autoclave sterilization. The design of the US probe holder will allow for easy removal and application of a sterile condom onto the probe. The new prototype will also support semi-automatic saturation biopsy through the registration of a prostate atlas and a biopsy scheme with real-time segmented prostate.

We are training the segmentation module on patient data. Real-time segmentation will cope with the movement of the prostate during the biopsy procedure, through 2D contour registration and subsequent tracking of the prostate. The image analysis software will also include techniques to handle prostate deformation and will pave the way for the next level of autonomy.
